# Injustice Without Evidence: The Unique Role of Conspiracy Theories in Social Justice Research

**DOI:** 10.1007/s11211-021-00376-x

**Published:** 2021-09-28

**Authors:** Jan-Willem van Prooijen

**Affiliations:** 1 Department of Experimental and Applied Psychology, VU Amsterdam, van der Boechorststraat 7, 1081BT Amsterdam, The Netherlands; 2 The Netherlands Institute for the Study of Crime and Law Enforcement, Amsterdam, The Netherlands; 3 Department of Criminal Law and Criminology, Maastricht University, Maastricht, The Netherlands

**Keywords:** Conspiracy theories, Social justice, Evidence, Existential threat, Group allegiances

## Abstract

Conspiracy theories are widespread and have a profound impact on society. The present contribution proposes that conspiracy theories are explanatory narratives that necessarily contain justice judgments, as they include attributions of blame and accusations of unethical or criminal conduct. Conspiratorial narratives also are mental simulations, however, and may elicit genuine feelings of injustice also without evidence of actual malpractice. Indeed, conspiracy theories sometimes describe unfair events that are unlikely to have occurred, unethical authorities that might not actually exist, and so on. Here I propose two complementary processes that stimulate belief in evidence-free conspiracy theories: (1) Existential threats instigate biased mental processing and motivated reasoning, that jointly promote an alternative perception of reality; and (2) group allegiances shape how people perceive, interpret, and remember facts to highlight the immoral qualities of competing outgroups. Due to these processes, conspiracy theories elicit a set of distinct reactions such as poor health choices and rejection of science. Moreover, evidence-free conspiracy theories require interventions beyond traditional approaches to install justice principles, such as debunking falsehoods and reducing polarized intergroup distinctions. I conclude that the scientific study of conspiracy theories is part of, and has a unique place in, social justice research.

## The Unique Role of Conspiracy Theories in Social Justice Research

When an angry crowd of Trump supporters stormed Capitol Hill on 6 January 2021, it was clear that they were motivated by a deeply felt sense of injustice. Indeed, several of the topics traditionally studied in social justice research appeared to be driving the rioters, including perceived distributive injustice (i.e., a belief that the outcome of the 2020 Presidential election was unfair) and procedural injustice (i.e., a belief that the election results were achieved through unfair procedures). A closer look reveals, however, that these traditional conceptualizations of social justice are insufficient to fully comprehend the rioters’ feelings and actions: They were substantially motivated by conspiracy theories, broadly defined as explanatory beliefs that a group of actors collude in secret to pursue malevolent goals (for overviews, see Butter & Knight, 2020; Douglas et al., 2019; Van Prooijen, 2018a, 2020; Van Prooijen & Douglas, 2018). In fact, many of the rioters were part of the so-called Qanon movement that promulgates a set of conspiracy theories alleging that President Trump is fighting a secret war against an evil Democratic “deep-state”, and that includes accusations of satanic rituals and sexual abuse of children among prominent members of the Democratic party.

In the present contribution, I propose that such conspiracy theories are narratives that necessarily contain justice judgments, as they involve attributions of blame and accusations of unethical, deceptive, or criminal behavior. But, an additional property of conspiracy theories is that they are mental simulations of events that might, or might not, actually have occurred. While actual corruption and conspiracies do occur, in politics (e.g., Watergate; the Iran-contra scandal), business (e.g., the Volkswagen Diesel scandal), the media (e.g., the BBC misleading Princess Diana to give an interview), and many other settings, also without any evidence of true malpractice people experience a genuine sense of injustice through conspiratorial accusations. Conspiracy theories may include convicted beliefs in falsehoods such as that the corona virus is a bioweapon, that climate change is a hoax, or that the Earth is flat. Instead, conspiracy theories are largely based on group allegiances: The mere existence of an outgroup that one distrusts or despises is enough for people to make unfounded assumptions of severely unethical behavior taking place in secret (Van Prooijen, 2020). In the following, I first describe more specifically how justice judgments are related with conspiracy theories. After that, I will illuminate what psychological processes stimulate belief in evidence-free conspiracy theories, and how this gives conspiracy beliefs a unique place in social justice research.

## Conspiracy Theories and Judgments of Injustice

The narrative structure of conspiracy theories suggests a close relationship with judgments of injustice. Specifically, any conspiracy theory contains five critical ingredients (Van Prooijen & Van Vugt, 2018; Van Prooijen, 2018a). These include (1) *patterns*: Assumptions of cause-and-effect relationships between stimuli; (2) *agency*: Assumptions that the events that one seeks to explain were caused on purpose, by intentional and deliberate actors; (3) *groups*: Conspiracies by definition consist of multiple actors that form a coalition and coordinate their actions; (4) *threat*: The assumed conspiracy intends to cause harm or at least is deceptive; and (5) *secrecy*: The assumed conspiracy acts in the shadows, making conspiracy theories difficult to invalidate. The link with judgments of injustice is underscored particularly by the notions that the assumed conspirators are deliberate (*agency*) and harmful or deceptive (*threat*). As such, conspiracy theories assign moral responsibility to a group of actors for assumed malpractice, and hence include judgments of moral wrongness and/or blame (e.g., Alicke, 2000; Malle, 2021). Many conspiracy theories assume severe criminal behavior on part of the conspirators, which would lead to long prison sentences if they were held legally accountable. Moreover, conspiracy beliefs predict a range of emotions commonly associated with justice judgments (for overviews, see Butter & Knight, 2020; Douglas et al., 2019; Van Prooijen & Douglas, 2018).

The level of injustice that is assumed to take place arguably is more severe in some conspiracy theories than others. Some conspiracy theories describe unambiguously criminal behavior such as murder (e.g., conspiracy theories that the corona virus was spread deliberately to kill elderly people; or, that the 9/11 terrorist strikes were an inside job of the US government), sexual abuse (e.g., Qanon), or fraud (e.g., conspiracy theories that climate change is a hoax by scientists to acquire government funding). Other conspiracy theories may not accuse the perpetrators of causing direct physical or financial harm to members of the community (e.g., beliefs that the moon landings were filmed in a TV studio; beliefs that the government hides evidence for the existence of intelligent alien life). Still, also the latter conspiracy theories include judgments of injustice, as they explicitly imply that the assumed conspirators are dishonest, and deliberately mislead the community to pursue a secret agenda.

The notion that conspiracy theories consist of judgments of injustice suggests that they are conceptually different from distrust. Feelings of distrust—while empirically related with justice judgments (Van den Bos et al., 1998)—are not justice judgments per se, as they do not necessarily include judgments or moral wrongness or attributions of blame. Instead, distrust refers to a generalized feeling that one prefers to avoid vulnerability toward a particular actor or group (Spadaro et al., 2020), and one can easily distrust an individual or group without concrete accusations of unethical behavior. While certainly there are parallels between institutional distrust and conspiracy theories, for instance, in their effects on social relationships (Van Prooijen et al., 2022), these two constructs are empirically and conceptually distinct (Abalakina-Paap et al., 1999; Pummerer et al., 2021).

One unique property of conspiracy theories is that they are mental simulations in which people “connect dots” in order to construct a coherent—and often spectacular—narrative of injustice that might, or might not, have occurred. Put differently, conspiracy theories are a set of assumptions how stimuli might be interrelated, to explain events such as election outcomes, terrorist strikes, a pandemic, and so forth. These assumptions may be mistaken, however, and many common conspiracy theories indeed are quite evidence-free (e.g., Qanon; Flat Earth beliefs; beliefs that climate change is a hoax; and so on). Sometimes it even happens that clearly fictional narratives turn into conspiracy theories that people truly believe: For instance, some people nowadays believe the conspiracy theory described in the novel “the Da Vinci Code” (Newheiser et al., 2011; see also Van Prooijen et al., 2021).

These issues are related to the *secrecy* ingredient of conspiracy theories: The conspiracy that is assumed to commit unethical behavior operates out of plain sight, and its existence or actions therefore have not been established with certainty. Scientific definitions do not assume conspiracy theories to be false per se: They are about *unproven* allegations of collusion. If an actual conspiracy gets exposed (e.g., the FIFA corruption scandal), it is not a “theory” anymore but an example of actual conspiracy formation. This implies that conspiracy theories are narratives constructed under conditions of informational uncertainty, and are based on people’s unproven assumptions of unethical behaviors that are covered up. These assumptions can be based on a vast range of stimuli that may include factual or plausible information, but also one’s own imagination, intuition, or falsehoods that perceivers encounter in personal conversations, on social media, and the Internet.

The notion that conspiracy theories are unproven, and often evidence-free, hold implications for the contribution of this emerging research field to social justice research more broadly. Specifically, the insight that people can experience a true sense of injustice based on imaginary events expands on many “traditional” studies that have focused on justice judgments based their appraisals of realistic events. For instance, the field of distributive justice has discovered that through social comparison people may evaluate the outcomes they get out of social exchanges (e.g., salaries) as fair or unfair, to the extent that they elicit feelings of relative deprivation (Stouffer et al., 1949; for an overview, see Smith et al., 2012) or inequity (Adams, 1965). Likewise, the field of procedural justice has emphasized that the fairness of decision-making procedures weight more heavily on their sense of justice than the outcomes of a decision-making process (Lind & Tyler, 1988; Thibaut & Walker, 1975), and that people care about receiving “voice” in decision-making procedures (Folger, 1977; Lind et al., 1990). Other domains of justice focus on the perceived fairness of punishment (Carlsmith & Darley, 2008; Van Prooijen, 2018b), justice-based responses to innocent victims (Lerner, 1980; see also Hafer & Bégue, 2005), or moral judgments and moral conviction across situations (Graham et al., 2009; Skitka, 2010). Most studies in these research domains focused on how people form justice judgments in the context of realistic everyday life situations, involving actual resource distributions and decision-making procedures, and truly existing decision-making authorities, offenders, victims, morally relevant situations, and so on.

While conspiracy theories reflect a deep-rooted and genuinely felt sense of injustice, they often are based on events that are highly unlikely to have taken place, or even demonstrably did not take place. Many US citizens continue to believe that the 2020 presidential elections were stolen, despite that all court trials challenging the election result were dismissed due to a lack of evidence, and recounts in multiple battleground states did not change the results. Other conspiracy theories rely even less on reality or facts. Some conspiracy theories attribute severely unethical behavior to authorities that are unlikely to actually exist: For instance, a common belief among members of extremist groups is the existence of an evil and secret world government, and that known political leaders are merely string puppets of this hidden powerful organization (Bartlett & Miller, 2010).

Moreover, some conspiracy theories portray unjust events that are unlikely to actually have taken place, involving offenders and victims that are unlikely to exist. Edgar Maddison Welch was convinced that a Democratic pedophile network kept innocent children hostage in the basement of a pizza restaurant; upon violently trying to release the children, however, he discovered that the restaurant did not have a basement to begin with. Yet, the belief in organized pedophile networks among high-ranked Democrats (and the notion that Democratic elites use the word “pizza” as codeword to order children for sexual abuse) continues to be a leading theme in the Qanon movement. Finally (and arguably most disturbingly), some conspiracy theories deny the existence of actual victims. This occurs in so-called crisis-actor conspiracy theories: In the aftermath of the Sandy Hook shootings, a common conspiracy theory was that the event was staged and the murdered children never existed. This has led conspiracy theorists to harass the parents of some of the deceased children (for an overview of these and many other evidence-free conspiracy theories, see Butter & Knight, 2020).

In the following, I introduce the emerging research domain of belief in conspiracy theories in more detail, and particularly focus on the question how conspiracy theories enable deep feelings of injustice without evidence that actual injustice has occurred. I propose that two interrelated psychological processes stimulate belief in evidence-free conspiracy theories: (1) an epistemic sense-making process that is triggered by existential threats; and (2) group allegiances, in particular an evolved tendency to blame existential threats on the covert actions of hostile coalitions.

## Existential Threats

One common insight is that conspiracy theories flourish particularly in societal crisis situations such as a pandemic, a terrorist strike, a war, a revolution, a natural disaster, or the unexpected death of a celebrity (Pipes, 1997; Van Prooijen & Douglas, 2017). This insight is consistent with the Uncertainty Management Model, stating that in uncertain situations people are more concerned about perceived justice or injustice than in certain situations (Van den Bos & Lind, 2002). Central in societal crisis situations are *existential threats*, which is a composite term for the feelings of anxiety and uncertainty that people experience when they perceive distressing events as threatening to one’s values, one’s way of life, or one’s existence. The relevance of these feelings for conspiracy theories are articulated in the Existential Threat Model of conspiracy theories (Van Prooijen, 2020), which proposes that anxiety- or uncertainty-provoking events stimulate an epistemic sense-making process that forms the basis of conspiracy thinking. Evidence for instance reveals that belief in conspiracy theories increases following threats to control (Van Prooijen & Acker, 2015; Whitson & Galinsky, 2008), threats to the status quo (Jolley et al., 2018), impactful and negative societal events (Van Prooijen & Van Dijk, 2014), experiences of social exclusion (Poon et al., 2020), relative deprivation and discrimination (Crocker et al., 1999; Van Prooijen et al., 2018b), feelings of uncertainty (Newheiser et al., 2011; Van Prooijen & Jostmann, 2013) and attitudinal ambivalence (van Harreveld et al., 2014).

The notion that feelings of existential threat stimulates epistemic sense-making processes is consistent with broader models about the role of stress in human psychology. These models have illuminated that stress stimulates not only specific appraisals, but also a broader search for meaning that people for instance may find in religion or spirituality (Park, 2010). Of importance for the present purposes, the epistemic sense-making processes following existential threats are based on a vigilant mindset that is especially prone to overestimate other’s hostile intent, which is consistent with social justice theories about self-interest and blame. Having incomplete information about other’s actions is already sufficient to increase the “myth of self-interest”, meaning that people “fill in the blanks” by overrating the extent to which others act in their self-interest (Vuolevi & Van Lange, 2010). Moreover, the Culpable Control Model proposes that harmful events stimulate an information processing style that is focused on ascribing moral responsibility and blame (Alicke, 2000). Here, I propose that these epistemic sense-making processes following existential threats are susceptible to biased automatic mental processes and motivated reasoning, which jointly promote a distorted perception of reality.

### Biased Mental Processes

The vigilant mindset underlying epistemic sense-making (over-)activates at least two basic processes of the mind to understand one’s social environment. The first relevant mental process is pattern perception: The mind’s property to establish meaningful relationships between stimuli, and to establish cause-effect relationships. While pattern perception is highly functional to identify truly existing patterns in one’s social environment, people sometimes also perceive *illusory* patterns: Meaningful relationships between stimuli that actually are random. Illusory pattern perception is for instance common among habitual gamblers (Wilke et al., 2014). Existential threats have been found to increase illusory pattern perception. For instance, inducing a lack of control increases people’s tendency to perceive patterns in snowy pictures, illusory correlations in stock market information, superstition, and conspiracy beliefs (Whitson & Galinski, 2008; see also van Harreveld et al., 2014). Illusory pattern perception is one of the key ingredients of evidence-free conspiracy theories, which also “connect dots” between events, persons, and places, that might well be a coincidence. Seeing patterns in random coin toss outcomes, or in the abstract paintings of Jackson Pollock, predicts belief in implausible, and even experimenter-designed conspiracy theories (van Prooijen et al., 2018a). Moreover, conspiracy beliefs are associated with a belief that spurious correlations reflect actual causal relationships (e.g., chocolate consumption is correlated with the number of Nobel prize winners in a country; see van der Wal et al., 2018).

The second relevant mental process is agency detection: The mind’s property to identify intentionality in social relationships. Agency detection is functional in social relationships, as recognizing other’s intentions may help people to distinguish friends from foes, or to infer important social norms in a given situation. Sometimes people may engage in hyperactive agency detection, however, and infer agency where none exists. Existential threats activate these agency detection mechanisms: For instance, uncertainty increases perceptions of agency in random events, and fuels supernatural beliefs (Valdesolo & Graham, 2014). Assumptions of agency also are a necessary ingredient of conspiracy theories, as they portray distressing societal events (e.g., an election loss, a pandemic) as the result of an intentional and evil scheme by powerful conspirators. Hyperactive agency detection indeed is associated with conspiracy beliefs: Detecting agency in the classic Heider and Simmel footage predicts increased belief in conspiracy theories (Douglas et al., 2016). Moreover, conspiracy beliefs are associated with teleological thinking, that is, attributions of purpose and cause to natural events and entities (Wagner-Egger et al., 2018).

Existential threats hence contribute to evidence-free conspiracy theories by stimulating two mental processes that distort people’s perception of reality: Perceiving patterns in stimuli that are random, and seeing agency where none exists. Such a distorted perception of reality is likely to also manifest itself in other judgmental domains. A broad range of studies support this notion. Belief in conspiracy theories is positively related with intuitive thinking yet negatively with analytic thinking (Swami et al., 2014), and is closely coupled with a range of non-evidentiary beliefs such as witchcraft, PSI, spiritualism, superstition, and precognition (Darwin et al., 2011). Moreover, conspiracy beliefs are associated with an increased tendency to perceive nonsense statements as profound (“bullshit receptivity”; Pennycook et al., 2015), with an increased likelihood of committing the conjunction fallacy (Brotherton & French, 2014), and with stereotyping (Imhoff & Bruder, 2014). Together, these findings are consistent with the idea that following existential threats, conspiracy theories emerge from a range of automatic mental sense-making processes that increases the likelihood of bias in people’s perception of reality.

### Motivated Reasoning

While the automatic mental processes described above may help explain how people initially form evidence-free conspiratorial beliefs following existential threats, more controlled, deliberative processes likely are at play in subsequently maintaining conspiracy theories. Quite often, conspiracy theories are carefully constructed narratives that are based on an extensive search for information, and combine factual information and scientific knowledge with misinformation and falsehoods (Butter & Knight, 2020). Once people are committed to the notion that a conspiracy must have been responsible for the events causing feelings of existential threat, they may rationalize their beliefs through a motivated reasoning process that selectively interprets information as evidence supporting their conspiracy theory. These rationalization processes are related with classic insights on cognitive dissonance, which have illuminated the mental processes through which people can maintain their beliefs even when their ideas and predictions are disconfirmed (e.g., doomsday cults that maintain their beliefs even after seeing their end-of-the-world prophecies fail; Festinger et al., 1956). Moreover, these rationalization processes resemble tunnel vision in criminal investigations, which is responsible for many wrongful convictions of innocent people (Findley & Scott, 2006).

After endorsing a particular conspiracy theory, it is common for people to regularly encounter information from authoritative sources that is incompatible with their theory. For instance, people who believe that the corona pandemic is a hoax—constructed by powerful elites to control regular citizens—inevitably will encounter news reports of patients suffering and dying from Covid-19 at Intensive Care Units. Such discrepant information may sometimes lead people to abandon, or at least modify, their conspiracy theory, but not necessarily so. Through motivated reasoning people may question the reliability or authority of the sources (e.g., assuming the news reports were designed by the conspiracy to mislead citizens), or downplay the relevance of the information in ways that enable them to uphold their conspiracy theories (e.g., “People also die of seasonal flu”). Furthermore, people have the tendency to interpret the scientific consensus on relevant issues as matching their existing conspiracy beliefs. For instance, people who believe that climate change is a hoax are more likely than others to believe that the scientific evidence for climate change is inconclusive (Kahan et al., 2011).

Moreover, the present digital era provides unprecedented opportunity to find some evidence for almost any belief, and get into contact with like-minded others who validate one’s conspiracy theories. A simple Internet search can bring people to well-designed and professionally appearing websites full of “evidence” that the Earth is flat, and that scientists have been lying about that for centuries (i.e., the “Flat Earth Movement”). Likewise, people searching for information about vaccines easily end up at anti-vax websites full of misinformation, for instance proclaiming that vaccines cause autism, and listing all the “dangerous” substances that vaccines contain.^1^ Due to their commitment to find evidence for a conspiracy theory, people do not weigh conflicting sources of evidence equally. Specifically, people hold lower evidentiary standards for information supporting a preferred conclusion than for information not supporting a preferred conclusion (Epley & Gilovich, 2016). The result of these motivated reasoning processes is a confirmation of one’s initial beliefs, perpetuating belief in conspiracy theories even if there is no genuine evidence to support them.

## Group Allegiances

The above section has illuminated how existential threat instigates biased mental processes that enable people to accept a distorted perception of reality, and that through motivated reasoning they maintain (and may even get more convicted of) that alternative reality. Here, I argue that one critical ingredient is missing in the processes described so far: The role of group allegiances. Conspiracies by definition consist of multiple hostile people coordinating their actions (i.e., “lone wolfs” do exist, but are not a conspiracy). These groups can be diverse and may be corporate (e.g., pharmaceutical companies in anti-vaccine conspiracy theories) and governmental (e.g., the US government hiding evidence for the existence of alien life); also, they may pertain to stigmatized minority groups in society (e.g., “Eurabia” conspiracy theories, asserting there is a Muslim conspiracy to Islamize Europe).

Such group allegiances help explain why existential threats do not lead to conspiracy theories all the time, or among all citizens. When the 9/11 terrorist attacks happened, it not only stimulated many conspiracy theories alleging that the attacks were an ‘inside job’ by the US government: In the months after the attacks, then-President George W. Bush also had the highest approval ratings ever recorded for a US president. Accordingly, feeling out of control may stimulate conspiracy theories in some situations (Van Prooijen & Acker, 2015; Whitson & Galinsky, 2008), but it may also increase people’s support for the government in other situations (Kay et al., 2008). Moreover, while motivated reasoning processes may reinforce conspiracy theories, System Justification Theory describes how similar motivated reasoning processes may lead disadvantaged people to see the political system that they live in as fair (Jost et al., 2004).

The existential threat model asserts that distressing societal events stimulate conspiracy theories only when people can blame the events on the secret actions of a salient antagonistic outgroup (Van Prooijen, 2020). As such, many conspiracy theories blame distressing societal events on groups that perceivers considered to be hostile to begin with, for example, due to ideological differences or prejudice. Accordingly, conspiracy theories about the 9/11 attacks are more common among Democrats (given that there was a Republican administration in office when it happened), while conspiracy theories about Obama’s birth certificate are more common among Republicans (Uscinski et al., 2016). A similar process can be seen after election loss (for a theoretical framework, see Uscinski & Parent, 2014): Following the 2016 US presidential election result, a common conspiracy theory among Democrats was that Donald Trump had colluded with the Russians and Wikileaks to win the elections; but following the 2020 US presidential election result, a common conspiracy theory among Republicans was that Democrats had stolen the elections through foul play.

### Adaptive Conspiracism

To understand the role of group allegiances in conspiracy theories, I propose that one needs to integrate basic insights of evolutionary psychology into the psychological processes underlying conspiracy beliefs. The Adaptive Conspiracism Hypothesis has illuminated that the human susceptibility to conspiracy theories is part of a set of mental predispositions that evolved to cope with hostile groups and coalitions (Van Prooijen & Van Vugt, 2018; for related arguments, see Haselton & Nettle, 2006; McKay & Dennett, 2009; Raihani & Bell, 2018). Throughout history, human beings have faced a real and pertinent threat to their health, safety, and reproductive opportunities: The dangers of other humans forming coalitions against them. Lethal intergroup conflict and coalitionary killings have significantly shaped the process of natural selection in humans (Bowles, 2009). The basic idea of the Adaptive Conspiracism Hypothesis, then, is that the mental faculties that promote suspicious feelings toward other groups—forming the basis of conspiracy theories, as well as a range of related human responses—evolved as an adaptive solution to the real problem of hostile coalitions. A propensity to easily be suspicious of potentially hostile coalitions would have given ancestral humans a selection advantage, by stimulating them to anticipate on possible lethal violence in a timely fashion (e.g., by migrating into safety, committing a pre-emptive strike, setting up a solid defense system, and so on).

How is the reality of frequent intergroup violence in an ancestral environment relevant to understand present-day conspiracy theories that people endorse without evidence? Two complementary issues are of importance here. The first is that also in an ancestral environment, it can be adaptive to be overly suspicious toward other groups even if this implies frequent mistakes in the process. Error management theory has proposed that when the costs of false-negatives (e.g., not detecting a conspiracy that actually exists) are unequal to the costs of false-positive (e.g., detecting a conspiracy that does not actually exist), people will evolve to make the less costly type of mistake. This evolutionary process explains a broad range of present-day human biases (Haselton & Nettle, 2006). In the case of conspiracy theories, it is plausible that the costs of false-negatives vastly exceeded the costs of false-positives in an ancestral environment. False-positives would for instance deprive ancestral human groups of possible allies, or stimulate unnecessary migration from a safe environment. False-negatives, however, would make groups of people vulnerable to deadly surprise attacks, and even genocide. Being overly susceptible to conspiracy theories hence could be adaptive even when it would imply many false conspiratorial allegations.

The second issue is that we no longer live in an ancestral environment, but in a modern environment. Scholars have noted that many evolved traits can be understood as an evolutionary mismatch: The human environment changed at a faster pace than human brains, and therefore, many evolved predispositions that were once functional in an ancestral environment are no longer functional, and often even dysfunctional, in a modern environment. A classic example of a mismatch is the human appetite for sweetness: In ancestral environments sweet cravings would stimulate consumption of nutritious foods found in nature (berries, yam, honey); yet, in a modern environment it leads to overconsumption of sweetened manufactured products (e.g., soda, candy) causing dental problems, obesity, and diabetes (for an overview, see Li et al., 2018). The human susceptibility to conspiracy theories may be a similar mismatch: In an ancestral environment being overly suspicious of unknown, and potentially powerful tribes in the vicinity could be life-saving. But in a modern environment, the same mental faculties produce evidence-free conspiracy theories that are irrelevant for one’s personal safety and reproductive opportunities, and may even be dysfunctional by disrupting society or stimulating poor health choices (e.g., unfounded beliefs that elections were stolen; beliefs that pharmaceutical companies tampered with vaccines; Van Prooijen & Van Vugt, 2018).

Consistent with the Adaptive Conspiracism Hypothesis, conspiracy theories have occurred across times and cultures. They are common in all cultures examined so far, including traditional societies that still live like hunter-gatherers (Van Prooijen & Van Vugt, 2018; West & Sanders, 2003). Moreover, they have been widespread throughout history, including ancient Rome, Greek mythology, the early and late Middle Ages, and the entire twentieth century (e.g., Butter & Knight, 2020; Pagan, 2008; Uscinski & Parent, 2014; Van Prooijen & Douglas, 2017). The most crucial hypothesis emerging from the evolutionary argument, however, is that there should be an intimate relationship between conspiracy beliefs and conflict between groups: If the human susceptibility to conspiracy theories evolved to protect against the dangers of hostile coalitions, it follows that the mental mechanisms that produce conspiracy theories should be especially sensitive to environmental cues suggesting possible intergroup conflict.

### Intergroup Conflict

When groups prepare for conflict with adversarial groups—which may include ideological conflicts, resource-based conflicts, or violent conflicts—two complementary psychological processes typically take place: First, ingroup favoritism increases, and second, people start perceiving the competing outgroup as symbolically or realistically threatening (Tajfel & Turner, 1979). This combination of ingroup favoritism and perceived outgroup threat puts normative pressure on group members to support narratives that favor their own group and highlight the immoral qualities of the competing outgroup. A strong predictor of conspiracy beliefs is descriptive norms, that is, the belief that other ingroup members endorse a set of conspiracy beliefs as well (Cookson et al., 2021). This normative pressure has effects ranging beyond motivated reasoning only: Research on political partisanship suggests that political affiliations influence how people perceive facts. For example, both Democrats and Republicans suffer from the “objectivity illusion”, that is, the belief that their own party’s beliefs are based on objective information while the opposing party’s beliefs are based on a biased assessment of information (Schwalbe et al., 2020). Moreover, people tend to reconstruct factual information (through biased perceptions, evaluations, and memory) in such a way to support and protect their political identity (Van Bavel & Pereira, 2018).

Consistent with the Adaptive Conspiracism Hypothesis, ample evidence underscores that the combination of increased ingroup favoritism and outgroup threat predicts belief in conspiracy theories. For instance, collective narcissism is defined as an exaggerated belief in the greatness of one’s ingroup (e.g., feelings of national superiority), and is associated with intergroup conflict. Such collective narcissism is a robust predictor of belief in conspiracy theories, focusing on ethnic groups subject to prejudice (e.g., Jewish conspiracy theories; Golec de Zavala & Cichocka, 2012), or competing political parties during an election campaign (Golec de Zavala & Federico, 2018). Furthermore, studies in Indonesia have examined Muslims’ conspiracy theories that blame domestic terrorist strikes on the hidden and deliberate actions of Western countries. One study manipulated the salience of participants’ Muslim identity, while also manipulating a newspaper article describing how threatening or unthreatening Western countries are to the Islamic world. Results revealed that the outgroup threat manipulation shaped belief in these conspiracy theories, but only when participants’ Muslim identity was made salient (Mashuri & Zaduqisti, 2015).

Also, international geopolitical conflicts are associated with conspiracy beliefs. One set of studies took place in the spring of 2019, at the height of the US-China trade war. The studies drew participant samples from both the US and China, and focused on mutual intergroup conspiracy theories: US participants were asked to what extent they believed that Chinese institutions were conspiring against America, and Chinese participants were asked to what extent they believed that American institutions were conspiring against China. Results revealed that such intergroup conspiracy theories were stronger in the Chinese than in the US samples. These differences were attributable to cultural variables that allow for hierarchy in society, specifically power distance values and vertical collectivism. The basic idea is that these cultural dimensions imply a norm to prioritize group goals over personal goals, which increases people’s sensitivity to cues suggesting that another group threatens their own. Supporting this notion, the relationships of these cultural variables with intergroup conspiracy beliefs were mediated by collective narcissism and perceived outgroup threat, suggesting that the observed cultural effects are due to differences in perceived intergroup conflict (Van Prooijen & Song, 2021). In sum, intergroup conflict is a major predictor of belief in conspiracy theories.

## Implications and Conclusions

This contribution sought to illuminate that conspiracy theories include justice judgments, as conspiratorial narratives ascribe blame and make assumptions of unethical behavior committed by other groups. A particularly interesting quality of conspiracy theories is that they enable people to genuinely experience a strong sense of injustice without evidence that any malpractice actually or even plausibly took place. What drives people’s belief in such evidence-free conspiracy theories? Here, I have proposed two complementary processes to answer this question. First, existential threats instigate biased mental processes, increasing the likelihood that people accept distorted views of reality in which they assign blame in unrealistic ways. Once people have accepted such an alternative reality, subsequent motivated reasoning processes allow them to justify, and therefore maintain or even amplify these views. Second, group allegiances promote evidence-free conspiracy theories. As part of an evolutionary heritage of an ancestral past—where false-negatives in gauging the likelihood of deadly intergroup conflict were especially costly—group allegiances shape how people perceive, interpret, and remember facts to support a narrative that highlights the deceptive and immoral qualities of competing outgroups. These processes increase people’s susceptibility to evidence-free conspiracy theories particularly when they perceive how a valued ingroup is in conflict with an outgroup.

The insights discussed here have two broad theoretical implications for the study of social justice. First, while many prior social justice studies have examined justice judgments as emotional responses to, or subjective appraisals of, more realistic events, the literature on conspiracy theories clarifies that people also may form severe judgments of injustice through (sometimes far-fetched) assumptions of events unlikely to have taken place. This suggests relatively complex psychological dynamics, and introduces difficult challenges to modify such beliefs. For instance, while existing authorities may increase feelings of procedural justice among followers by using decision-making procedures that includes a range of procedural justice criteria (Leventhal, 1980), mitigating unfounded beliefs that authorities run an organized network to perform satanic rituals or sexually molest children is quite another issue. Second, group allegiances are a justice cue in and of itself. While it has been widely recognized that group allegiances are important to understand a range of justice judgments, which are closely coupled with social identity concerns (e.g., Lind & Tyler, 1988; Tyler & Lind, 1992), the field of conspiracy theories suggests that the mere existence of an antagonistic outgroup is sufficient for people to draw inferences of severe injustices being covered up by that group—also without concrete evidence that members of the outgroup actually have behaved unfairly. Together, these two implications give conspiracy theories a unique place in social justice research.

The link between conspiracy theories and social justice judgments is also reflected in the effects of conspiracy theories on human beliefs and behaviors. In particular, many responses to “traditional” conceptualizations of social justice and conspiracy theories converge. For instance, procedural justice judgments have been associated with a range of human responses such as trust in authorities, positive and negative emotions, protest behaviors, deference to rules, and so on (Tyler & Lind, 1992). Moreover, both perceived procedural injustice, and strong moral convictions, are associated with radicalization and disregard for the rule of law (Van den Bos, 2018; Skitka, 2010). Similar responses have been observed among people who believe conspiracy theories: Conspiracy beliefs predict distrust (Abalakina-Paap et al., 1999; Pummerer et al., 2021), collective action (particularly non-normative forms of collective action, such as vandalizing, attacking police officers, harassing people online, and so on; see Imhoff et al., 2021; Rottweiler & Gill, 2020), a willingness to commit minor forms of crime (Jolley et al., 2019), extremism, populism, and radicalization (Bartlett & Miller, 2010; Krouwel et al., 2017; Silva et al., 2017; Van Prooijen et al., 2015), and a range of human emotions (for overviews, see Douglas et al., 2019; Van Prooijen & Douglas, 2018). Such convergence supports one of the basic premises of this contribution, namely that conspiracy theories necessarily include judgments of injustice.

Evidence-free conspiracy theories also elicit a set of unique responses, however, that can be understood only when taking the alternative reality that believers have accepted into account. For instance, belief in conspiracy theories predict a range of beliefs and behaviors that hurt perceivers’ own health. Conspiracy theories about vaccines lower vaccination intentions (Jolley & Douglas, 2014), and conspiracy beliefs about the Covid-19 pandemic decreases physical distancing (Pummerer et al., 2021). Studies in South Africa have revealed that AIDS conspiracy beliefs (e.g., beliefs that AIDS has been deliberately created by humans) substantially lower condom use among both men and women (Grebe & Nattrass, 2012). Moreover, conspiracy theories undermine social policy by calling the validity of scientific knowledge into question. People who believe that climate change is a hoax—fabricated by scientists to acquire research funding—are unlikely to support policy to reduce global warming, or to reduce their own carbon footprints (Van der Linden, 2015). Finally, conspiracy beliefs stimulate forms of radical action that are not easily explained by traditional conceptualizations of social justice: For example, conspiracy beliefs that link the Covid-19 pandemic to 5G radiation predicts justification of destroying telecommunication masts (Jolley & Paterson, 2020).

Conspiracy theories also raise new challenges for interventions designed to increase feelings of fairness among the public. Feelings of injustice caused by truly occurring events are commonly addressed through tailored behavioral interventions, which may include changes in how outcomes are distributed (e.g., reducing inequality), attention for procedural justice criteria in decision-making, punishment of norm violators, compensation or other forms of justice restoration for victims, and so on. While these interventions may help reducing conspiracy theories in some settings (e.g., Van Prooijen, 2019), it is likely that evidence-free conspiracy theories also require different tools. In a polarized social setting characterized by strong intergroup conflict, members of competing outgroups may continue to elicit conspiracy theories despite their best efforts to be fair. Debunking falsehoods contributes to reducing conspiracy theories (Orosz et al., 2016), as does shifting people’s motivations to a concern for accuracy (Pennycook et al., 2021). Moreover, interventions to break down rigid intergroup allegiances, and increase reconciliation, may reduce conspiracy theories (Van Prooijen, 2020).

At the same time, it should be noted that research on effective interventions to reduce belief in evidence-free conspiracy theories is still in its infancy (Van Prooijen & Douglas, 2018). Moreover, some possible interventions may raise new social, moral, and practical dilemmas. For instance, it is very well possible that recent initiatives from social media companies (e.g., Twitter; Facebook) to remove misinformation and “fake news” from their forums is effective in reducing the spread of evidence-free conspiracy theories. Yet, such interventions do raise important new questions, such as who gets to decide what type of information is or is not allowed in public discourse, what the criteria are for classifying information as “false”, and what the implications are for citizens’ basic constitutional rights (e.g., freedom of speech). Indeed, history has shown many examples of organized misconduct by legitimate authorities that would seem implausible at first, yet turned out true (e.g., the Snowden surveillance revelations). These dilemmas suggest many intriguing novel hypotheses for social justice researchers to explore.

To conclude, this contribution was designed to highlight how the emerging research domain of belief in conspiracy theories fits into social justice research. Conspiracy theories necessarily contain justice judgments, as they involve attributions of moral responsibility, blame, and accusations of unethical, and often criminal conduct. Moreover, conspiracy theories underscore that people’s own imagination can promote justice appraisals: Many conspiracy beliefs are based on an alternative reality that consists of falsehoods, enabling a genuine sense of injustice about events that might not have taken place, authorities that might not exist, and so on. Far-fetched conspiracy theories can have a real impact on society, however, as underscored by events such as the Capitol Hill riots, anti-lockdown protests, anti-vaccination campaigns on social media, electorally successful populist movements around the world, death threats of public figures, and so on. These issues suggest that the field of conspiracy theories raises a set of complex new questions that require extensive attention of social justice researchers, practitioners, and policy-makers.

## Data Availability

Not applicable.

## Footnotes

1 Note that these lists are misleading as any substance has a “Threshold of Toxicological Concern” above which it is toxic for humans. For comparison: Vaccines also contain water, which by the same token is dangerous as people regularly drown in the ocean.
